# Supplementary material for: Novel Driver Strength Index highlights important cancer genes in TCGA PanCanAtlas patients
Source: PeerJ. 2022 Aug 11;10:e13860. doi: 10.7717/peerj.13860 (PMC9375969; doi:10.7717/peerj.13860)
Supplement: Supplemental Information 2 [file peerj-10-13860-s002.zip › COHORTS/genes plots/age/2021_8_16_14_9_distribution_cohorts_males_75-79.pdf]

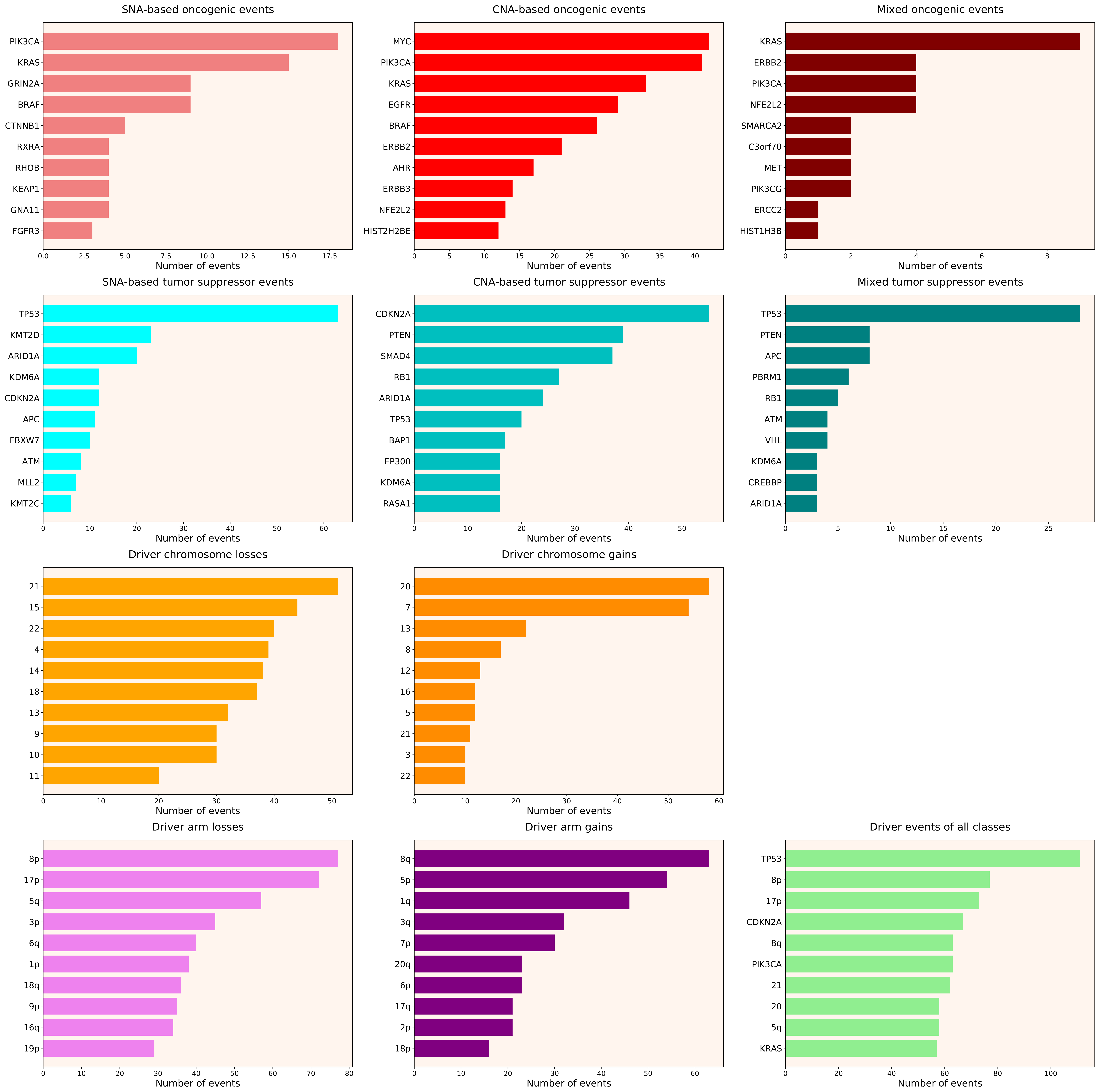
SNA-based oncogenic events
PIK3CA
KRAS
GRIN2A
BRAF
CTNNB1
RXRA
RHOB
KEAP1
GNA11
FGFR3
0.0 2.5 5.0 7.5 10.0 12.5 15.0 17.5
Number of events
CNA-based oncogenic events
MYC
PIK3CA
KRAS
EGFR
BRAF
ERBB2
AHR
ERBB3
NFE2L2
HIST2H2BE
0 5 10 15 20 25 30 35 40
Number of events
Mixed oncogenic events
KRAS
ERBB2
PIK3CA
NFE2L2
SMARCA2
C3orf70
MET
PIK3CG
ERCC2
HIST1H3B
0 2 4 6 8
Number of events
SNA-based tumor suppressor events
TP53
KMT2D
ARID1A
KDM6A
CDKN2A
APC
FBXW7
ATM
MLL2
KMT2C
0 10 20 30 40 50 60
Number of events
CNA-based tumor suppressor events
CDKN2A
PTEN
SMAD4
RB1
ARID1A
TP53
BAP1
EP300
KDM6A
RASA1
0 10 20 30 40 50
Number of events
Mixed tumor suppressor events
TP53
PTEN
APC
PBRM1
RB1
ATM
VHL
KDM6A
CREBBP
ARID1A
0 5 10 15 20 25
Number of events
Driver chromosome losses
21
15
22
4
14
18
13
9
10
11
0 10 20 30 40 50
Number of events
Driver chromosome gains
20
7
13
8
12
16
5
21
3
22
0 10 20 30 40 50 60
Number of events
Driver arm losses
8p
17p
5q
3p
6q
1p
18q
9p
16q
19p
0 10 20 30 40 50 60 70 80
Number of events
Driver arm gains
8q
5p
1q
3q
7p
20q
6p
17q
2p
18p
0 10 20 30 40 50 60
Number of events
Driver events of all classes
TP53
8p
17p
CDKN2A
8q
PIK3CA
21
20
5q
KRAS
0 20 40 60 80 100
Number of events
